# Ascorbic acid metabolism and functions

**DOI:** 10.1093/jxb/erae143

**Published:** 2024-04-26

**Authors:** Patricia L Conklin, Christine H Foyer, Robert D Hancock, Takahiro Ishikawa, Nicholas Smirnoff

**Affiliations:** Biological Sciences Department, Bowers Hall Rm 240, SUNY Cortland, Cortland, NY 13045, USA; School of Biosciences, College of Life and Environmental Sciences, University of Birmingham, Edgbaston B15 2TT, UK; Cell and Molecular Sciences, The James Hutton Institute, Invergowrie, Dundee, DD2 5DA, UK; Shimane University, 1060 Nishikawatsu, Matsue, Shimane 690-8504, Japan; Biosciences, Faculty of Health and Life Sciences, University of Exeter, Exeter EX4 4QD, UK

**Keywords:** Ascorbic acid, ascorbate peroxidase, ascorbate oxidase, dehydroascorbate reductase, l, -galactose, GDP-, l, -galactose phosphorylase, glutathione reductase, mannose, monodehydroascorbate reductase, oxalic acid, redox signalling, tartaric acid, threonic acid, uORF

## Abstract

**This Special Issue was assembled to mark the 25th anniversary of the proposal of the** **d** **-mannose/** **l** **-galactose (Smirnoff–Wheeler) ascorbate biosynthesis pathway in plants (** **Wheeler *et al.*, 1998** **). The issue aims to assess the current state of knowledge and to identify outstanding questions about ascorbate metabolism and functions in plants.**

Ascorbate is also known as vitamin C because humans and some other animals have lost biosynthetic capacity and require a dietary source. It is present in all green plants, reaching the highest concentrations in photosynthetic tissue. Also, the fruits of some species such as *Myrciaria dubia* (camu-camu) (Vargas *et al.*, 2024) and *Terminalia ferdinandiana* (Kakadu plum) (Phan *et al.*, 2021) contain very high ascorbate for unknown reasons: perhaps to attract fruit dispersers or act as an antioxidant preservative. The reviews in this Special Issue cover all aspects of ascorbate metabolism and function, providing a snapshot of current knowledge and pointers to unanswered questions. Ascorbate in plants has garnered attention because of its antioxidant role in scavenging reactive oxygen species (ROS) such as hydrogen peroxide and radicals (Fig. 1). This antioxidant activity links ascorbate to the wider antioxidant system and redox signalling (Foyer and Kunert, 2024). The link between ascorbate accumulation and light is related to its photoprotective roles in photosynthesis, both in scavenging hydrogen peroxide and as a substrate for violaxanthin de-epoxidase, a photoprotective enzyme. The roles of ascorbate in iron trafficking and in preventing inactivation of 2-oxoglutarate-dependent dioxygenases (2-ODDs) have received far less attention in plants than in biomedical research. 2-ODDs catalyse many reactions required for synthesis of hormones, specialized metabolites, and extracellular proteins. Most famously, impaired hydroxylation of proline and lysine residues in collagen is a prominent symptom of scurvy, the ascorbate deficiency disease. More recently, there has been a focus in mammals on its role in influencing the activity of DNA and histone demethylases thereby influencing epigenetic control (Smirnoff and Wheeler, 2024).

**Fig. 1.**
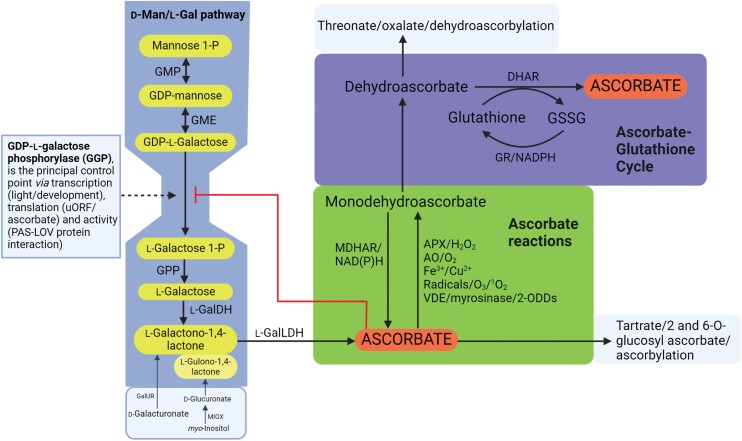
An outline of the biosynthesis and reactions of ascorbate in plants covered in this Special Issue. The diagram illustrates the d-mannose/l-galactose biosynthetic pathway and the proposed main flux controlling step (GDP-l-galactose phosphorylase). Uronic acids can be converted to ascorbate if made available by feeding or by overexpression of relevant enzymes, and d-galacturonate derived from pectin breakdown could provide a minor route in wild-type plants. The green box includes the biochemical reactions of ascorbate enabling it to act as an antioxidant/radical scavenger, enzyme substrate (VDE), enzyme cofactor (myrosinase), or a chaperone preventing inactivation by overoxidation of active site Fe in 2-ODDs. The primary oxidation product, monodehydroascorbate, is a relatively stable radical which is regenerated to ascorbate or disproportionates to dehydroascorbate (DHA). Ascorbate is regenerated from DHA in the ascorbate–glutathione cycle (blue box) which links ascorbate to the wider thiol system. Other ascorbate-derived compounds are shown. Proteins and small molecules can be (dehydro)ascorbylated, but little is known about these products. Abbreviations: AO, ascorbate oxidase; APX, ascorbate peroxidase; DHAR, dehydroascorbate reductase; l-GalDH, l-galactose dehydrogenase; l-GalLDH, l-galactono-1,4-lactone dehydrogenase; d-GalUR, galacturonate reductase; GME, GDP-mannose-3’,5’-epimerase; GMP, GDP-mannose pyrophosphorylase; GPP, l-galactose 1-P phosphatase; GR, glutathione reductase; GSSG, oxidized glutathione; MDHAR, monodehydroascorbate reductase; MIOX, *myo*-inositol oxygenase; 2-ODD, 2-oxoglutarate-dependent dioxygenase; VDE, violaxanthin de-epoxidase.

## Ascorbate: a complex chemistry and evolutionary history

There is a diversity of ascorbate biosynthesis pathways across different groups of organisms (Wheeler *et al.*, 2015; Smirnoff, 2018; Duque *et al.*, 2022). The immediate precursor is an aldono-1,4-lactone. In animals, l-gulono-1,4-lactone is derived from UDP-d-glucuronate but, in contrast, in the plant the d-Man/l-Gal pathway, it is derived from l-galactono-1,4-lactone via GDP-mannose (Fig. 1). Photosynthetic protists utilize UDP-galacturonate but convert it to l-galactono-1,4-lactone and then to ascorbate. In animals, there is a suggestion that some invertebrates have an unidentified pathway different from the standard pathway (Duque *et al.*, 2022). Furthermore, fungi make erythroascorbate (a C_5_ ascorbate analogue) from d-arabinose (Loewus, 1999). The relatively complex chemistry of ascorbate provides multiple biochemical roles (Fig. 1). Most noted is removal of H_2_O_2_ catalysed by plant-specific ascorbate peroxidases (APXs). Ascorbate also neutralizes a range of free radicals and reduces higher oxidation states of Fe and Cu, enabling roles in preserving the activity of Fe-containing 2-oxoglutarate dioxygenases and potentially in Fe (and Cu) mobilization.

## GDP-l-galactose phosphorylase controls ascorbate biosynthesis

Smirnoff and Wheeler (2024) review the history of research on ascorbate biosynthesis and describe the d-Man/l-Gal (Smirnoff–Wheeler pathway) (Fig. 1). They highlight evidence that GDP-l-galactose phosphorylase (GGP), the first step unique to ascorbate synthesis, exerts the greatest degree of control over the rate of synthesis and ascorbate content. Its expression is controlled at transcriptional and translational levels via an upstream ORF (uORF) and by light-dependent interaction with a PAS-LOV photoreceptor protein. The emerging details of these control mechanisms are reviewed by Baldet *et al.* (2024). uORFs tend to control translation of regulatory genes but also biosynthetic enzymes. This is proposed to occur via interaction of metabolites and translated uORF peptides associated with the ribosome (van der Horst *et al.*, 2020), and could enable feedback repression of ascorbate synthesis by ascorbate. The critical discovery that GGP translation is controlled by a uORF (Laing *et al.*, 2015) has enabled production of high ascorbate plants by gene editing (Baldet *et al.*, 2024), and efforts to understand translational control of GGP will provide a model for this form of control in plants. Further insights into the control of the pathway will be facilitated by structural studies of the enzymes and, towards this goal, Vargas *et al.* (2024) provide crystal structures of three of the d-Man/l-Gal pathway enzymes. Considering the central role of GGP, we await its structure, particularly in view of its interaction with PAS-LOV and possibly with other pathway enzymes (Fenech *et al.*, 2021). Maruta *et al.* (2024) provide new insights into the evolution of the whole ascorbate system (including APXs and recycling enzymes) since ascorbate concentration increases from green algae through to angiosperms. Importantly, they propose protection against the reactivity of DHA as an important factor in accumulation of high amounts of ascorbate (see Box 1).

Considering the diversity of ascorbate biosynthesis pathways noted above, the possibility of pathway diversity within green plants, including use of d-glucuronate and d-galacturonate, is reviewed by Quinoñes *et al*. (2024). These compounds are readily converted to ascorbate if applied as their methyl esters (Loewus and Kelly, 1961; Davey *et al.*, 1999). Furthermore, overexpression of d-galacturonate reductase (Agius *et al.*, 2003) and *myo*-inositol oxygenase (MIOX) (Quiñones *et al.*, 2024) increases ascorbate. MIOX is likely to work by increasing d-glucuronate production. These results raise the question of the role of uronic acids as ascorbate precursors in wild-type plants. Given that labelled *myo*-inositol is not converted to ascorbate (Loewus, 1999), this pathway is unlikely to be significant in wild-type plants. However, if sufficient d-galacturonate is available, for example from pectin breakdown in ripening fruit, it could be converted to ascorbate alongside the d-Man/l-Gal pathway (Hancock *et al.*, 2007; Badejo *et al.*, 2011; Alegre *et al.*, 2020). It should be noted that, at least in Arabidopsis, knockout of d-Man/l-Gal-specific enzymes causes lack of ascorbate and growth arrest after germination, indicating that the alternative pathways are quantitatively unimportant (Lim *et al.*, 2016; Fenech *et al.*, 2021). The extensive ethyl methanesulfonate (EMS) mutagenesis screen carried in Arabidopsis only identified enzymes involved in the d-Man/l-Gal pathway and VTC3, a predicted protein phosphatase/kinase (Conklin *et al.*, 2013).

## Many proposed functions and still much to learn

Identification of ascorbate-deficient Arabidopsis *vtc* mutants (Conklin *et al.*, 2000) was central to identifying the biosynthetic pathway, but also provided material for investigating ascorbate functions. Low ascorbate mutants are more susceptible to (photo)oxidative stresses, explaining the light responsiveness of ascorbate synthesis in some species. However, effects on growth and development are less clear cut (Box 1). The most striking feature of the transcriptomes of *vtc* mutants is an increase in pathogen defence-related transcripts and a corresponding increase in basal resistance to biotrophic pathogens (Pastori *et al.*, 2003; Barth *et al.*, 2004; Conklin and Barth, 2004; Mukherjee *et al.*, 2010). Interestingly, somatic embryogenesis is enhanced in a *vtc* mutant (Becker *et al.*, 2014), lending support to a role for ascorbate status in redox control of development (Foyer and Kunert, 2024). More ascorbate is therefore not always good (Box 1), emphasizing the requirement for control of its biosynthesis via GGP.

The antioxidant function of ascorbate has a strong focus in photosynthetic organisms because of the presence of a plant-specific APX (a type 1 haem peroxidase) enzyme family present in the cytosol, chloroplasts, mitochondria, and peroxisomes which functions along with thiol peroxidases to remove hydrogen peroxide. The functions and regulatory features of APX, including post-translational modifications, are reviewed in three articles (Corpas *et al.*, 2024; Foyer and Kunert, 2024; Yoshimura and Ishikawa, 2024). The oxidized forms of ascorbate, monodehydroascorbate (MDHA), and dehydroascorbate (DHA), generated from its reaction with hydrogen peroxide and radicals, are reduced to ascorbate by NAD(P)H-dependent MDHA reductases and glutathione-dependent DHA reductases (Fig. 1). Regeneration of ascorbate by the ascorbate–glutathione (Foyer–Halliwell–Asada) cycle links ascorbate oxidation to the redox state of glutathione and the wider range of thiol proteins involved in redox signalling. The background to the ascorbate–glutathione cycle and recent advances in the properties of the enzymes and roles of the cycle in signalling are reviewed by Foyer and Kunert (2024). The possible roles in influencing the thiol redox state of the nucleus are discussed.

Ascorbate is present in the apoplast where it tends to be more oxidized (higher proportion of DHA) than in the intracellular pool, partly because of the presence of a plant-specific enzyme ascorbate oxidase (AO) in the cell wall. Apoplastic DHA is transported into the cytosol via an unidentified transporter and can also be converted to oxalate and threonate. These reactions are described by Ford *et al.* (2024) along with intracellular enzymatic formation of tartrate, which is formed by a narrower range of species, including grapes. The functions of ascorbate breakdown in cell wall loosening and defence are discussed by Ford *et al.* (2024). Intracellular calcium oxalate crystals (in specialized vacuoles) derived from ascorbate are formed *in situ* (Kostman *et al.*, 2001), although it should be noted that oxalate is formed from glyoxylate in rice (Yu *et al.*, 2010). Related to the oxidative breakdown of ascorbate, Mellidou and Kanellis (2024) review the functions of apoplastic AO. Early studies of AO showed high expression in rapidly expanding tissues, suggesting a role in growth and, more recently, investigation of overexpression lines and mutants with decreased AO activity suggest relatively subtle effects on development.

## Ascorbate as a paradigm for control of metabolism by environmental factors

The advances over the last 25 years in understanding ascorbate biosynthesis, breakdown, and functions in plants provide a basis for addressing the exciting emerging questions outlined in Box 1. Understanding ascorbate biosynthesis will assist production of crop plants with enhanced nutritional value and stress resistance. More generally, the d-Man/l-Gal pathway provides a paradigm for understanding the control of metabolism via integration of transcriptional, translational, and protein–protein interaction-mediated mechanisms.

Box 1. **Outstanding questions**

**The control of ascorbate synthesis by GGP.** Multiple lines of evidence indicate that GDP-l-galactose phosphorylase (GGP/AtVTC2 and AtVTC5) activity controls ascorbate synthesis. An upstream ORF (uORF) in the 5’-untranslated region (5’-UTR) encodes a conserved peptide, and its mutation increases GGP translation and ascorbate accumulation (Baldet *et al.*, 2024). Is this peptide synthesized and what is its role in repressing translation? Does ascorbate or a proxy for ascorbate status interact with the uORF/peptide to provide feedback repression of GGP translation?

**What is the role of protein–protein interaction in GGP activity?** GGP interacts with a blue light receptor-like PAS-LOV protein. This inhibits enzyme activity, and dissociation is caused by blue light, suggesting a mechanism for increased ascorbate accumulation in the light (Aarabi *et al.*, 2023; Baldet *et al.*, 2024). Photosynthesis-sourced signals and changes in GGP transcripts are proposed to mediate the light response. What is the relative importance of these mechanisms and what is the photosynthetic signal? GGP may interact with other enzymes in the d-Man/l-Gal pathway: does this occur *in vivo*, and does it form a metabolon to influence pathway flux? Structural studies will cast light on this question (Vargas *et al.*, 2024).

**Why is increased GGP expression in pollen deleterious?** Increased expression of GGP in tomato (by mutating the uORF) (Deslous *et al.*, 2021) and Arabidopsis (by pollen-specific GGP overexpression) (Weigand *et al.*, 2023) causes sterility via impairment of pollen development and growth. The reason for this effect is not known although ascorbate is not increased in GGP-overexpressing Arabidopsis pollen. Possible mechanisms include direct toxicity of excess ascorbate/DHA or its breakdown products, hydroxyl radical generation via the Fenton reaction, or disruption to GDP-mannose supply for pollen growth.

**What is the function of VTC3?** VTC3 is a predicted dual-function protein kinase/phosphatase unique to plants. *vtc3* mutants in *Arabidopsis thaliana* and *Physcomitrium patens* have decreased ascorbate, but its function is unknown.

**What are the ‘basal’ functions of ascorbate?** Knockout of ascorbate-specific biosynthetic enzymes prevents ascorbate synthesis and results in arrest of seedling growth after germination while Arabidopsis *vtc* mutants with ~20% wild-type ascorbate can grow. Which processes fail between 0% and 20% ascorbate? We suggest that potential roles in Fe mobilization and as a cofactor/chaperone of 2-oxoglutarate-dependent dioxygenases with multiple functions will become more apparent, along with increased oxidative stress.

**What is the function of ascorbate oxidase?** Mutants with decreased AO activity do not reveal a specific function for AO. What would be the phenotype of AO null mutants?

**Which proteins mediate intra- and intercellular ascorbate and DHA transport?** A chloroplast envelope and, recently, a vacuolar ascorbate transporter, have been identified. We do not know which proteins mediate ascorbate and DHA transport across the plasma membrane and other organelles.

**Can we build an ascorbate biosensor?** Glutathione redox and H_2_O_2_ biosensors are proving useful for investigating cell- and organelle-specific events, and an ascorbate biosensor would add to this armoury. This would, for example, enable investigation of the proposed lack of ascorbate in the root quiescent centre (Kerk and Feldman, 1995). An ascorbate-sensing protein which de-represses the *Escherichia coli* ascorbate utilization regulon (Garces *et al.*, 2008) could provide a starting point.
